# Assessing the Alignment Between the Humpty Dumpty Fall Scale and Fall Risk Nursing Diagnosis in Pediatric Patients: A Retrospective ROC Curve Analysis

**DOI:** 10.3390/healthcare13141748

**Published:** 2025-07-19

**Authors:** Manuele Cesare, Fabio D’Agostino, Deborah Hill-Rodriguez, Danielle Altares Sarik, Antonello Cocchieri

**Affiliations:** 1 A. Gemelli IRCCS University Hospital Foundation, Section of Hygiene, Department of Health Science and Public Health, Catholic University of the Sacred Heart, 00168 Rome, Italy; antonello.cocchieri@policlinicogemelli.it; 2 Department of Medicine, Saint Camillus International University of Health Sciences, 00131 Rome, Italy; fabio.dagostino@unicamillus.org; 3 Nicklaus Children’s Hospital, Miami, FL 33155, USA; deborah.hill-rodriguez@nicklaushealth.org (D.H.-R.); danielle.sarik@nicklaushealth.org (D.A.S.)

**Keywords:** risk assessment, accidental falls, standardized nursing terminology, nursing diagnosis, Humpty Dumpty Fall Scale, ROC curve

## Abstract

**Background/Objectives**: Falls in hospitalized pediatric patients are frequent and can lead to serious complications and increased healthcare costs. Nurses typically assess fall risk using structured tools such as the Humpty Dumpty Fall Scale (HDFS), alongside nursing diagnoses such as Fall risk ND, which are based on clinical reasoning. However, the degree of alignment between the HDFS and the nursing reasoning-based diagnostic approach in assessing fall risk remains unclear. This study aims to assess the alignment between the HDFS and Fall risk ND in identifying fall risk among hospitalized pediatric patients. **Methods**: A retrospective observational study was conducted in a tertiary pediatric hospital in Italy, including all pediatric patients admitted in 2022. Fall risk was assessed within 24 h from hospital admission using two approaches, the HDFS (risk identified with the standard cutoff, score ≥ 12) and Fall risk ND, based on the nurse’s clinical reasoning and recorded through the PAI*ped* clinical nursing information system. Discriminative performance was analyzed using receiver operating characteristic curve analysis. The area under the curve (AUC), sensitivity, specificity, positive predictive value (PPV), and negative predictive value (NPV) were calculated. A confusion matrix evaluated classification performance at the cutoff (≥12). **Results**: Among 2086 inpatients, 80.9% had a recorded Fall risk ND. Of the 1853 patients assessed with the HDFS, 52.7% were classified as at risk (HDFS score ≥ 12). The HDFS showed low discriminative ability in detecting patients with a Fall risk ND (AUC = 0.568; 95% CI: 0.535−0.602). The PPV was high (85.1%), meaning that most patients identified as at risk by the HDFS were also judged to be at risk by nurses through Fall risk ND. However, the NPV was low (20.1%), indicating that many patients with low HDFS scores were still diagnosed with Fall risk ND by nurses. **Conclusions**: The HDFS shows limited ability to discriminate pediatric patients with Fall risk ND, capturing a risk profile that does not fully align with nursing clinical reasoning. This suggests that standardized tools and clinical reasoning address distinct yet complementary dimensions of fall risk assessment. Integrating the HDFS into a structured nursing diagnostic process—guided by clinical expertise and supported by continuous education—can strengthen the effectiveness of fall prevention strategies and enhance patient safety in pediatric settings.

## 1. Introduction

Falls are among the most frequent adverse events in hospitalized pediatric patients, with potential consequences ranging from minor injuries to serious harm, including fractures, increased length of stay, and elevated healthcare costs [1,2]. The pediatric population presents unique challenges in fall prevention due to developmental variability, impulsivity, and limited ability to perceive and avoid danger [2,3]. For this reason, early identification of children at risk is crucial for implementing timely and appropriate preventive measures and ensuring patient safety in hospital and community healthcare settings [4,5].

In clinical practice, nurses use two primary approaches to assess fall risk: structured tools and clinical reasoning through the identification of nursing diagnoses [6]. These strategies are complementary: while structured tools offer a standardization of the assessment, clinical reasoning enables individualized assessments. The literature emphasizes combining both to support more accurate fall risk identification [7,8].

Among the tools used for pediatric fall risk assessment, the Humpty Dumpty Fall Scale (HDFS) is one of the most adopted internationally [9]. The HDFS considers seven items and—based on a predefined cutoff—classifies patients as either at risk or at low risk for falling. In parallel, NDs related to fall risk—though labeled differently across standard nursing languages such as Clinical Care Classification (CCC) (e.g., Fall risk) and NANDA International (e.g., Risk for child falls)—are identified based on the nurse’s holistic assessment, knowledge of the phenomenon, and clinical experience [10,11]. Consequently, while the HDFS offers a standardized, quantitative approach to fall risk screening, ND reflects a broader clinical reasoning process and incorporates both clinical and contextual factors—such as patient-specific risks and environmental conditions—that may be missed by structured tools [12]. However, despite the widespread use of both the HDFS and Fall risk ND, these two approaches are often applied independently in clinical practice [6,8,13]. This siloed application has led to a lack of clarity regarding the level of concordance between the two methods in identifying patients at risk of falls. Specifically, it remains unclear whether the HDFS effectively discriminates those patients whom nurses, based on their clinical expertise, judge to be at risk—formalized through the identification of a Fall risk ND. Addressing this gap is essential, as understanding the alignment—or misalignment—between structured tools and clinical reasoning can inform more consistent and effective fall prevention strategies by enabling more accurate risk stratification and safer patient care.

This study aims to explore the relationship between structured fall risk assessment and nursing clinical reasoning in pediatric inpatients by examining the concordance between the HDFS and Fall risk ND. Specifically, this study has three specific objectives: (1) to evaluate the characteristics of pediatric inpatients based on the presence or absence of a Fall risk ND; (2) to describe patient characteristics according to fall risk classification as determined by the HDFS; and (3) to assess the concordance between the HDFS and Fall risk ND in the identification of fall risk, using ROC analysis and confusion matrix-based metrics to compare the two approaches.

## 2. Materials and Methods

### 2.1. Study Design and Setting

This retrospective, observational study was conducted in a tertiary pediatric hospital in Italy. Data were collected from the electronic health records of all hospitalized pediatric patients over a one-year period (January–December 2022). This study was reported in accordance with the STARD 2015 (Standards for Reporting Diagnostic Accuracy Studies) guidelines [14].

### 2.2. Participants

Inclusion criteria were patients (1) aged < 18 years and (2) hospitalized in any pediatric ward of the study hospital. Exclusion criteria included patients discharged within 24 h from hospital admission, as such hospitalizations are comparable to day hospital admission [15].

### 2.3. Variables and Data Collection

The following patient-related primary variables were included in the analysis and grouped into three main categories:

- Demographic and clinical variables.

These included patient age, gender, number of comorbidities, presence of chronic conditions, diagnosis-related group weight (DRGw)—used as an indicator of medical complexity [16]—and length of stay (LOS). These data were collected from the Hospital Discharge Register (HDR), the official administrative and clinical record compiled by all public and private hospitals in Italy [17].

- Nursing-related variables.

This category included the Fall risk ND, a standardized diagnosis assigned by nurses within the first 24 h of hospitalization. The ND was formulated using the Clinical Care Classification (CCC) System version 2.5, an internationally standardized nursing language adapted into Italian [18]. For descriptive purposes, all additional NDs and the corresponding nursing interventions (NIs) documented during the hospital stay were also considered, in order to contextualize the overall nursing complexity [12,15].

- Structured fall risk assessment.

Fall risk was also assessed using the HDFS, a standardized tool commonly adopted in pediatric inpatient settings. The HDFS includes seven weighted items (age, gender, diagnosis, cognitive impairment, environmental factors, response to surgery/sedation/anesthesia, and medication usage). Each item is assigned a weighted score, resulting in a total score ranging from 7 to 23. Based on the original validation study by Hill-Rodriguez et al. [9], a score of 12 or higher on the HDFS is considered the standard cutoff for identifying patients at high risk of falls. This threshold was empirically established during the initial pilot testing phase, as 13 out of 38 patients who had fallen scored between 12 and 13. Therefore, a score of 12 was adopted as the “cut point” to define high risk, and it has since been retained in clinical applications due to its practical discriminative value. In this study, the HDFS score refers to the assessment performed by nurses within the first 24 h of hospital admission; it was considered only in cases where it had been recorded within the same time frame as the Fall risk ND. The study hospital holds a license for the clinical use of the HDFS.

Nursing-related variables and structured fall risk assessment were collected through an electronic nursing documentation system called the Neonatal and Pediatric Professional Assessment Instrument (PAI*ped*). In the study hospital, this system is an integral part of clinical practice and is routinely used by nurses to document nursing care in accordance with the steps of the nursing process [19]. The PAI*ped* is a clinical nursing information system that enables structured documentation of nursing assessments, NDs, NIs, and clinical risk scores, including the HDFS [20]. PAI*ped* incorporates a validated, semi-automated decision-support algorithm [21] that connects specific assessment findings—such as signs, symptoms, and risk factors—with potential NDs. As the nurse completes the digital assessment, the system proposes diagnostic suggestions based on predefined associations, thereby supporting—but not replacing—clinical reasoning. The nurse can then accept or reject these suggestions, maintaining full clinical autonomy in diagnostic decision-making [22]. Additional information on the structure and documentation features of the PAI*ped* system can be found elsewhere [20,22]. 

### 2.4. Ethical Considerations

Ethical approval for this study was obtained from the Ethics Committee of the Catholic University of the Sacred Heart (Protocol no. 0012915/24, ID 6752, approved 16 May 2024). Given the retrospective study design, general informed consent for data processing was obtained from parents or legal guardians during hospitalization and recorded in the electronic health record. Efforts were made to re-contact guardians via mailed letters and phone calls to provide detailed information about this study—including data anonymization, participant rights, and the option to withdraw consent— and to reconfirm their participation and consent. Patients were excluded if contact could not be established after three attempts within a three-month period. Written consent was also obtained from healthcare professionals whose clinical documentation was used, ensuring full respect for privacy, professional rights, and ethical standards in line with the Declaration of Helsinki and Good Clinical Practice guidelines. Data were securely stored in a password-protected database accessible only to authorized researchers, in compliance with data protection regulations.

### 2.5. Statistical Analysis

Descriptive statistics were used to summarize the demographic, clinical, and organizational characteristics of the sample. Continuous variables were reported as means with standard deviations (SDs) or medians with interquartile ranges (IQRs), depending on data distribution, and categorical variables were reported as frequencies and percentages. Normality of distribution was assessed using skewness and kurtosis (i.e., skew > 2 or kurtosis > 7 indicating non-normality) [23]. Group comparisons were performed using independent-sample *t*-tests or Mann–Whitney U tests for continuous variables and chi-squared tests for categorical variables. Levene’s test was used to assess homogeneity of variances; if violated (*p* ≤ 0.05), Welch’s *t*-test was applied.

To assess the discriminative performance of the HDFS in identifying patients with a Fall risk ND, a receiver operating characteristic [21] curve analysis was conducted, treating the presence of the ND (yes/no) as the reference standard. The ND was treated as binary (0 = absent, 1 = present). The area under the ROC curve (AUC) with 95% confidence intervals (CIs) was calculated. AUC values range from 0.5—indicating no discriminative power, equivalent to random chance—to 1.0, which represents perfect discrimination. Interpretation thresholds commonly used in diagnostic accuracy studies classify AUC values as follows: values of 0.90 or higher are considered excellent; values from 0.80 to 0.89 are good; 0.70 to 0.79 is fair; 0.60 to 0.69 is poor; and 0.50 to 0.59 indicates fail-level performance [24]. Interpretation should also consider the 95% CI, which reflects the degree of uncertainty around the estimate: a narrower CI indicates greater precision and reliability, whereas a wider CI suggests reduced confidence in the reported AUC value [25].

To further evaluate classification performance at the standard HDFS cutoff (≥12), a confusion matrix was generated. In this matrix, true positives (TP) were patients classified as at risk by both the HDFS and ND; false positives (FP) were classified as at risk by the HDFS but not assigned ND; false negatives (FN) were not classified as at risk by the HDFS but did receive ND; and true negatives (TN) were not identified as at risk by either method. From this, the positive predictive value (PPV = TP/[TP + FP]) and negative predictive value (NPV = TN/[TN + FN]) were calculated to explore the agreement between the HDFS and ND [26]. The PPV represented the proportion of patients identified as at risk by the HDFS who were also assigned a Fall risk ND, indicating the likelihood of true positive concordance. Conversely, the NPV indicated the proportion of patients not identified as at risk by the HDFS who also lacked a Fall risk ND, thus assessing the test’s ability to correctly identify true negatives [27]. All analyses were performed using IBM SPSS Statistics for macOS, Version 30. A *p*-value < 0.05 was considered statistically significant.

## 3. Results

### 3.1. Patient Characteristics According to the Presence of a Fall Risk ND

**Figure 1:**
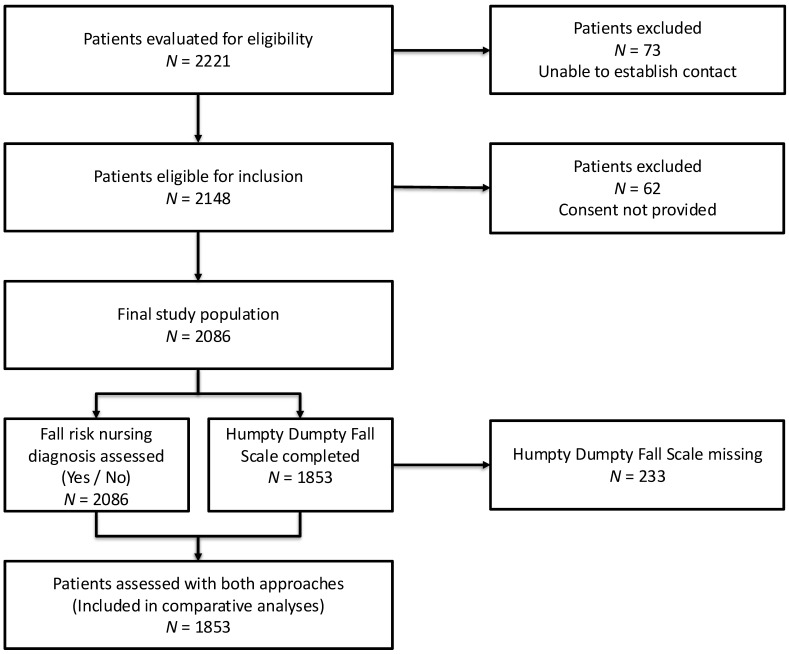
Flow diagram showing the number of pediatric inpatients evaluated for eligibility included in this study and assessed using Fall risk nursing diagnosis and/or the Humpty Dumpty Fall Scale within the first 24 h of admission.

A total of 2086 pediatric inpatients met the inclusion criteria and were included in the descriptive analysis. All patients were evaluated for the presence or absence of a Fall risk ND, whereas 1853 (88.8%) also had an HDFS score recorded within the first 24 h of admission. These 1853 cases were included in the comparative analyses between the two fall risk assessment approaches. Figure 1 illustrates the patient selection process and the distribution according to the availability of a Fall risk ND and HDFS assessment.

Among the final study population, 1689 patients (80.9%) had a documented Fall risk ND. Compared to those without this diagnosis, they were significantly younger and presented higher clinical and nursing complexity, including more comorbidities, chronic conditions, NDs, and NIs (all *p* < 0.001). They also showed increased healthcare resource use, reflected by a higher DRGw (*U* = 288,803.5, *p* < 0.001) and a longer LOS (*U* = 296,650.0, *p* < 0.001), despite similar medians. No differences were observed in gender distribution. As expected, patients in the Fall risk ND group also had higher scores on the HDFS (*p* < 0.001) (Table 1).

Regarding the HDFS classification, 52.7% (*n* = 976) of the 1853 assessed patients were classified as at risk for falls (HDFS ≥ 12). In contrast to those not at risk, these patients were significantly younger, more frequently male, and had more chronic conditions (all *p* < 0.001). They also exhibited lower DRGw (*p* < 0.001) and fewer NDs (*p* < 0.001). No significant differences emerged between the two groups in terms of the number of comorbidities, LOS, or NIs. As expected, HDFS scores were substantially higher in the at-risk group (*p* < 0.001) (Table 1). 

### 3.2. Patient Characteristics According to HDFS Items, Stratified by the Presence of a Fall Risk ND

To further explore the components underlying HDFS risk classification, descriptive statistics for each item of the scale were examined, stratified by the presence or absence of a Fall risk ND. The sample was predominantly composed of younger patients, with 73.7% under 13 years old and 27.0% under 3. Male patients represented 56.6% of the sample, with a similar distribution across both groups. Neurological diagnoses were significantly more common among patients with a Fall risk ND (*p* < 0.001), as were cognitive impairments, particularly being unaware of one’s limitations (*p* = 0.003). Environmental factors such as placement in high-risk settings (e.g., cribs, use of assistive devices) were also more prevalent in this group (*p* = 0.001), as was exposure to high-risk medications, including sedatives and narcotics (*p* = 0.002). In contrast, no significant differences were found in gender distribution, age groups, or recent exposure to surgery or anesthesia. Differences between patients with and without a Fall risk ND were observed across several HDFS items, particularly neurological status, cognitive function, environmental context, and medication use (Table 2).

### 3.3. Diagnostic Accuracy of the HDFS in Detecting Fall Risk ND

**Figure 2:**
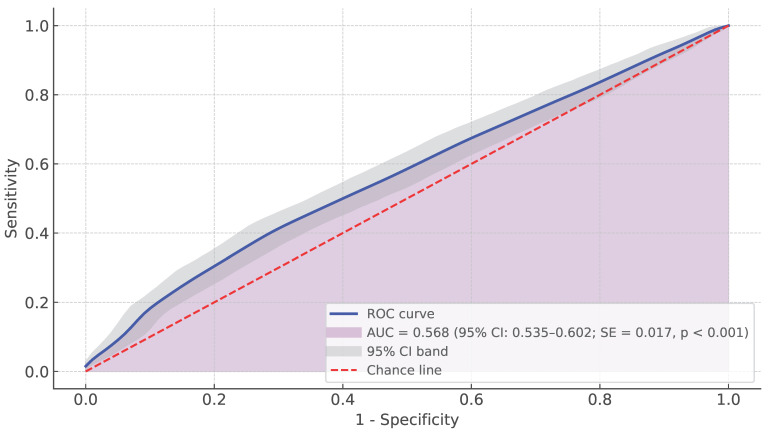
ROC curve. Note: The ROC curve illustrates the ability of the HDFS to distinguish between patients with and without a Fall risk ND. The Y-axis indicates sensitivity (true positive rate), and the X-axis represents 1 − specificity (false positive rate). A curve closer to the upper-left corner indicated better performance. Abbreviations: ROC, receiver operating characteristic; HDFS, Humpty Dumpty Fall Scale; AUC, area under the (ROC) curve; CI, confidence interval.

The ROC curve analysis demonstrated a limited ability of the HDFS to detect the presence of a Fall risk ND among the sample. The AUC was 0.568 (95% CI: 0.535−0.602; SE = 0.017, *p* < 0.001) (Figure 2), reflecting a fail-level discriminative performance [25], only marginally better than chance.

### 3.4. Predictive Validity of the HDFS in Identifying a Fall Risk ND

**Figure 3:**
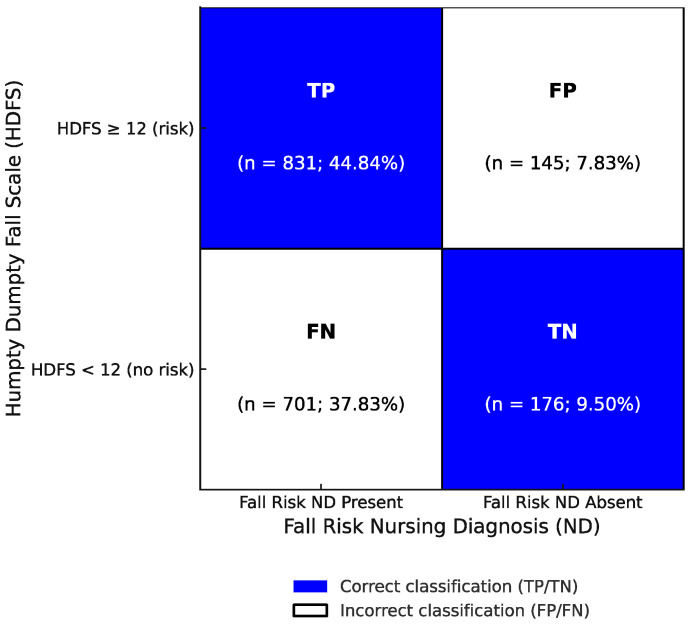
Confusion matrix comparing the HDFS classification (risk/no risk) against the presence or absence of a Fall risk ND. Note: The matrix includes the following patient counts: TP, true positives = 831 patients (classified as at risk by both the HDFS and Fall risk ND); FP, false positives = 145 patients (classified as at risk by the HDFS but not assigned a Fall risk ND); FN, false negatives = 701 patients (not classified as at risk by the HDFS but assigned a Fall risk ND); and TN, true negatives = 176 patients (not classified as at risk by either method). Correct classifications (TP and TN) are shown in blue, while misclassifications (FP and FN) are displayed in white. Percentages in each cell represent the proportion of patients among those with complete data for both the HDFS and a Fall risk ND (*N* = 1853). Abbreviations: HDFS, Humpty Dumpty Fall Scale; ND, nursing diagnosis; TP, true positives; FP, false positives; TN, true negatives; FN, false negatives; PPV, positive predictive value; NPV, negative predictive value.

The confusion matrix in Figure 3 illustrates the trade-off in predictive performance at an HDFS cutoff score ≥ 12. The PPV was high (85.1%), indicating that most patients identified as at risk by the HDFS also had a Fall risk ND, supporting its alignment with nursing clinical reasoning. Conversely, the NPV was low (20.1%), suggesting that a low HDFS score was often associated with patients who nonetheless received a Fall risk ND (Figure 3).

## 4. Discussion

This study explored the alignment between a structured fall risk assessment tool (i.e., the HDFS) and nursing clinical reasoning (i.e., Fall risk ND) in pediatric inpatients. Specifically, we analyzed the characteristics of patients classified as at risk through each method and assessed the ability of the HDFS to detect those judged to be at risk by nurses using the ND, through ROC analysis and classification metrics.

Our findings showed that patients with a Fall risk ND were generally younger, had more comorbidities and chronic conditions, and exhibited a higher DRGw—indicators of greater medical complexity. They also had a higher number of NDs and longer hospital stays. In parallel, patients classified as at risk according to the HDFS were also younger and more frequently affected by chronic conditions; however, their overall profile did not fully align with that of patients identified as at risk through Fall risk ND. Notably, patients flagged as at risk by the scale had a lower DRGw and fewer NDs than those deemed at risk based on clinical reasoning. At first glance, this may seem paradoxical, as one would expect patients identified as at risk to exhibit greater nursing complexity, reflected by a greater number of NDs [28]. This apparent inconsistency is likely attributable to the HDFS, which emphasizes specific fall-related risk factors—such as young age, male gender, or recent sedation—rather than broader clinical indicators that generally drive more complex nursing care.

Although our interpretation remains preliminary, the patient characteristics associated with fall risk through both approaches in our sample—structured assessment and clinical reasoning—appear broadly consistent with those reported in the literature, which highlights younger age, male gender, multiple chronic conditions, and neurological or cognitive impairments as key factors in pediatric fall risk [2,29,30,31]. Yet, the observed divergence in patient characteristics suggests that the HDFS and Fall risk ND may capture only partially overlapping—if not divergent—dimensions of fall risk. To better understand the alignment between the two methods, we conducted a ROC curve analysis to evaluate how well the HDFS detects patients judged at risk through clinical reasoning.

Our ROC curve analysis confirmed this misalignment, showing that the HDFS had limited ability to distinguish patients identified as at risk by Fall risk ND. Although the scale showed a high PPV—suggesting strong agreement when both strategies identified risk—the NPV was low. In other words, many patients deemed not at risk by the HDFS were nonetheless considered at risk by nurses using Fall risk ND. This finding confirms a conceptual misalignment between the structured approach of the HDFS and the clinical reasoning applied by nurses when formulating the Fall risk ND. While both strategies aim to identify fall risk, they operate on fundamentally different logics. The HDFS is a checklist-based tool that applies standardized criteria to generate a numeric score, ensuring consistent application across contexts [9,32]. In contrast, Fall risk ND results from clinical reasoning—an individualized process shaped by the nurse’s holistic understanding of the patient, contextual awareness, and professional judgment [10,33]. Previous research has similarly shown that structured assessment tools may not align with clinical judgment [34], which—like reasoning— draws on tacit knowledge and the nurse’s holistic interpretation of patient-specific data [35,36]. The HDFS emphasizes structured indicators—such as age and medication use—whereas the ND often reflects less quantifiable, experience-based insights. Consequently, although the scale includes important risk domains—such as cognitive impairment and environmental hazards—it may fail to capture subtler cues that nurses consider when formulating NDs. This highlights not only the intrinsic limits of relying solely on standardized tools to assess a multifactorial phenomenon like pediatric fall risk—a challenge well documented in the literature [5,7]—but also invites reflection on the nursing diagnostic process itself. In particular, the high prevalence of Fall risk NDs observed in our study may reflect a generally cautious approach adopted by nurses across care settings, possibly influenced by the preventive focus of nursing practice and heightened attention to patient safety [37]. This interpretation is supported by prevalence studies showing that Fall risk ND is consistently among the most frequently assigned NDs in hospital and pre-hospital settings, including pediatric and adult populations [12,38,39,40]. 

Although our study did not include actual fall events, the observed patterns raise important considerations regarding the potential for both over- and under-identification of risk across different methods. Under these circumstances, while tools like the HDFS offer consistency and structure, they may benefit from refinement to improve sensitivity, specificity, and overall diagnostic accuracy [32,41]. At the same time, nursing clinical reasoning—though valuable—remains subject to variability [42,43], highlighting the need for integrated approaches that balance standardization with individualized assessment. Otherwise, there is a risk of misclassification: under-identification could result in missed preventive opportunities (underprotection), whereas over-identification may lead to excessive restrictions (overprotection). In the context of falls, the former risks missing vulnerable patients; the latter may unnecessarily restrict mobility or autonomy. Both scenarios can cause unnecessary resource use, psychological distress for families, and inflated healthcare costs [44,45,46]. This underscores the importance of continuous monitoring and calibration of fall prevention strategies to ensure that interventions remain proportionate to actual risk [6]. Based on our data, nurses can achieve this by triangulating multiple data sources—such as HDFS scores and NDs within clinical nursing decision support systems such as PAI*ped*—to enable a more comprehensive and balanced approach to risk stratification and to support tailored, evidence-informed interventions, particularly in pediatrics, where risk factors are dynamic and context-sensitive [4]. 

Notably, while the HDFS is designed to identify patients at low risk (score 7–11) and high risk of falling (score > 12), NDs—formulated through clinical reasoning—may reflect both moderate and high levels of risk. This difference in threshold criteria likely contributes to the observed discordance between the two approaches. Therefore, future studies should explore the concurrent validity of using HDFS and NDs together, to enhance the accuracy of fall risk identification and to inform more refined, individualized prevention strategies. 

This study presents several limitations that should be considered when interpreting the findings. First, this study compared two proxy measures (HDFS and Fall risk ND) without evaluating their predictive validity against actual fall events, as this was beyond the scope of the study. Therefore, metrics such as sensitivity and specificity should be interpreted with caution, as they reflect the level of concordance between a structured tool and a diagnostic reasoning—not true diagnostic performance based on real outcomes. Future studies should aim to include objective clinical outcomes—such as the actual occurrence of falls—to assess the predictive validity of both the HDFS and Fall risk ND. Second, Fall risk ND, while standardized through the CCC taxonomy and supported by the PAI*ped* algorithm, remains a product of clinical reasoning and may be subject to variability based on nurse knowledge, experience, and contextual factors. It is important to remember that the use of NDs is directly linked to the effect of clinical reasoning, a complex process shaped by experience, context, and institutional culture [42,43]. Even with the support of standardized systems like the PAI*ped*, diagnostic processes remain interpretive and subject to individual variability [47]. In this context, although the PAI*ped* algorithm provides evidence-based suggestions, nurses may choose to assign or omit a ND based on their professional judgment. It is worth noting that the PAI*ped* system has been routinely used by nurses at the study hospital since its implementation in 2016. Regular training sessions are provided by the hospital’s nursing directorate to promote correct and consistent use, as part of a broader initiative to improve the quality of nursing documentation [20]. Importantly, the algorithm is designed to minimize the risk of ND underestimation, whereas the overestimation of fall risk may be more frequently driven by human factors—such as heightened clinical caution or contextual influences—particularly in a pediatric population inherently vulnerable to falls due to developmental, behavioral, and clinical complexities [2,48]. Third, due to the retrospective study design, a proportion of patients lacked HDFS data because completion of the scale was not mandatory at the time of admission and depended on nurses’ discretion. This may have introduced a selection bias and reduced the sample size for some analyses. Fourth, although we hypothesized that the HDFS and Fall risk ND may capture complementary dimensions of fall risk, this remains a theoretical proposition that requires empirical validation in future studies. Our study was not designed to identify or analyze discordant patient profiles (e.g., HDFS-negative/Fall risk ND-positive) but rather to evaluate the overall concordance between two diagnostic strategies. Future research should explore these discordant subgroups more specifically, to validate the distinct clinical perspectives captured by each method. Finally, this study was conducted in a single tertiary pediatric hospital in Italy, which may limit the generalizability of the findings to other settings or healthcare systems. Future prospective study designs would allow for more systematic and complete data collection, reducing the risk of missing data and selection bias. Moreover, future research could employ multivariable models to explore the role of clinical and organizational factors influencing the assignment of NDs. 

### Implications for Nursing Practice, Research, Education, and Policy

This study underscores the importance of integrating structured assessment tools with nursing clinical reasoning in pediatric fall risk evaluation. For nursing practice, combining standardized scales like the HDFS with NDs may enhance decision-making and patient safety, since they address the same risk from complementary perspectives. In nursing research, our findings highlight the need to validate fall risk tools against both diagnostic reasoning and real outcomes. Educational programs should emphasize the development of diagnostic reasoning and the critical use of standardized instruments. From a policy perspective, clinical nursing information systems should support decision-making through evidence-based scales (e.g., the HDFS) and standardized nursing languages (e.g., NDs) to improve care quality and safety.

## 5. Conclusions

This study highlights the limited discriminative ability of the HDFS in identifying pediatric inpatients assigned a Fall risk ND, underscoring a mismatch between this structured scale score and clinical nursing reasoning. Given the multidimensional nature of fall risk, no single tool can fully capture its complexity across different clinical settings, including the pediatric one [5]—particularly in light of the low specificity demonstrated by currently available pediatric fall risk scales [41,49]. Therefore, integrating standardized tools like the HDFS with NDs (i.e., Fall risk ND) and other validated assessment instruments may enhance the accuracy of risk identification. Future research should continue in this direction. Ongoing training, the refinement of tools, and the adoption of a multimodal approach remain essential to support accurate clinical decision-making and improve pediatric patient safety.

## Figures and Tables

**Table 1:** Patient characteristics according to the presence of a Fall risk ND (*N* = 2086) and HDFS risk classification (*N* = 1853).

Variable	Descriptive Statistics									
	Patients Without Fall Risk ND (*N* = 397)		Patients with Fall Risk ND (*N* = 1689)		*p*-Value ^a^	Patients Not at Risk for Falls (HDFS < 12) (*N* = 877)		Patients with Fall Risk (HDFS ≥ 12) (*N* = 976)		*p*-Value ^a^
**Age** (years) (mean; SD)	9.25	5.91	7.80	5.85	<0.001	10.81	5.02	5.01	4.81	<0.001
**Gender** (*N*; %)					0.641					<0.001
Male	221	55.7	962	57.0		393	44.8	658	67.4	
Female	176	44.3	727	43.0		484	55.2	318	32.6	
**Comorbidities** (mean; SD)	1.64	0.94	1.89	1.21	<0.001	1.85	1.22	1.90	1.13	0.394
**Chronic conditions** (mean; SD)	0.75	0.92	1.02	1.05	<0.001	0.85	1.02	1.17	1.05	<0.001
**DRG weight** (median, IQR)	0.7350	0.54	0.7933	0.74	<0.001	0.8102	0.69	0.6807	0.54	<0.001
**LOS** (days) (median, IQR)	4.00	4	4.00	4	<0.001	4.00	5	5.00	4	0.670
**NDs** (*N* = 8504) (mean; SD)	2.40	1.92	4.47	2.72	<0.001	4.41	2.53	3.86	2.87	<0.001
**NIs** (*N* = 23720) (mean; SD)	10.63	2.56	11.54	2.86	<0.001	11.44	2.77	11.60	2.64	0.209
	(*N* = 321)		(*N* = 1532)							
**HDFS score** (*N* = 1853) (mean; SD)	11.37	2.32	12.02	2.61	<0.001	9.69	1.10	13.90	1.76	<0.001

Abbreviations: ND, nursing diagnosis; HDFS, Humpty Dumpty Fall Scale; SD, standard deviation; DRG, diagnosis-related group; LOS, length of stay; NIs, nursing interventions. ^a^ = chi-squared test for categorical variables; *t*-test or Mann–Whitney *U* test for continuous variables, as appropriate; Welch’s correction applied when Levene’s test indicated unequal variances (*p* ≤ 0.05).

**Table 2:** Patient characteristics according to HDFS items (*N* = 1853), stratified by the presence of a Fall risk ND.

HDFS Item	Descriptive Statistics				
	Patients Without Fall Risk ND (*N* = 321)		Patients with Fall Risk ND (*N* = 1532)		*p*-Value ^a^
	*N*	%	*N*	%	
**Age**					0.098
Less than 3 years old	77	19.4	424	27.7	
3 to less than 7 years old	67	20.9	339	22.1	
7 to less than 13 years old	75	23.4	384	25.1	
13 years and above	102	31.8	385	25.1	
**Gender**					0.660
Male	178	55.5	870	56.8	
Female	143	44.5	662	43.2	
**Diagnosis**					<0.001
Neurological diagnosis	61	19.0	422	27.5	
Alterations in oxygenation (respiratory diagnosis, dehydration, anemia, anorexia, syncope/dizziness, etc.)	15	4.7	53	3.5	
Psych/behavioral disorders	42	13.1	102	6.7	
Other diagnosis	203	63.2	955	62.3	
**Cognitive Impairments**					0.003
Not Aware of Limitations	50	15.6	366	23.9	
Forgets Limitations	32	10.0	161	10.5	
Oriented to Own Ability	239	74.5	1005	65.6	
**Environmental Factors**					0.001
History of falls or infant–toddler placed in bed	5	1.6	30	2.0	
Patient uses assistive devices or infant–toddler in crib or furniture/lighting (tripled room)	2	0.6	41	2.7	
Patient placed in bed	288	89.7	1402	91.5	
Outpatient area	26	8.1	59	3.9	
**Response to Surgery/Sedation/Anesthesia**					0.516
Within 24 h	36	11.2	154	10.1	
Within 48 h	4	1.2	32	2.1	
More than 48 h/none	281	87.5	1346	87.9	
**Medication Usage**					0.002
Multiple usage of sedatives (excluding ICU patients sedated and paralyzed), hypnotics, barbiturates, phenothiazines, antidepressants, laxatives/diuretics, narcotics	1	0.3	43	2.8	
One of the meds listed above	13	4.0	115	7.5	
Other medications/none	307	95.6	1374	89.7	

Abbreviations: HFDS, Humpty Dumpty Fall Scale. ^a^ = chi-squared test.

## Data Availability

The data presented in this study are available on request from the corresponding author due to restrictions (privacy, legal, and ethical reasons).

## Abbreviations

The following abbreviations are used in this manuscript:

AUC	Area Under the Curve
CCC	Clinical Care Classification
CI	Confidence Interval
DRG	Diagnosis-Related Group
FN	False Negative
FP	False Positive
HDFS	Humpty Dumpty Fall Scale
HDR	Hospital Discharge Register
IQR	Interquartile Range
LOS	Length of Stay
ND	Nursing Diagnosis
NI	Nursing Intervention
NPV	Negative Predictive Value
PAI*ped*	Neonatal and Pediatric Professional Assessment Instrument
PPV	Positive Predictive Value
ROC	Receiver Operating Characteristic
SD	Standard Deviation
STARD	Standards for Reporting Diagnostic Accuracy Studies
TN	True Negative
TP	True Positive
